# Quality of pharmacy services and regulatory compliance

**DOI:** 10.12669/pjms.325.11416

**Published:** 2016

**Authors:** Shaukat Ali Jawaid

A study by Syed Shaukat Muttaqi and colleagues on “Quality of Drug Stores, Storage practices and Regulatory compliance being published in this issue makes startling disclosures which are of course not unexpected. The investigators inspected one thousand three drug stores in Karachi excluding those located inside the Government run healthcare facilities and found that only 4.1%(41 drug stores) were compliant with the regulatory requirements. Only one hundred twenty four i.e. 12% had qualified persons working on the stores of which 33% were qualified pharmacists. Just four hundred drug stores had displayed drug sales licenses at the premises of which 282 had expired licenses which were not renewed. Ninety four of these stores were found to be selling vaccines without proper refrigerator and only 11.7% of these stores had the power backup for the refrigerator. Four hundred three stores were protected from the direct sunlight and just 54 which are 5.4% had air conditioning at the premises.^1^

If this is the state of affairs in a city like Karachi, what would be the situation in the smaller cities and towns is not difficult to imagine. Improper storage and transportation without cold chain results in decreasing the potency of these drugs and when these samples are picked up by the Drug Inspectors for quality control analysis, it is the drug manufacturers who are blamed for producing sub-standard drugs. There also seems to be no justification whatsoever in not getting the licenses renewed which also shows the poor performance of those entrusted with the task of ensuring compliance with the regulatory requirements.

In most of the poor developing low and middle income couriers it is the private pharmacies, chemist shops which are considered as a valuable resource for providing health advice and medicines to the community. However, the quality of service they provide leaves much to be desired. Smith and colleagues reviewed the quality of private pharmacy services in low and middle income countries.^2^ This systematic review identified numerous deficiencies in the quality of professional practices. In particular they highlighted the non-availability of pharmacists or trained personnel working at these pharmacies. They were of the view that if the standard of pharmacy practice has to improve and pharmacists have to contribute effectively in healthcare, barriers to the provision of quality care and the ways through which these hindrances can be eliminated needs to be identified and examined.

Another study on community pharmacy in Pakistan^3^ found that the average age of attendants at the pharmacy shops was between 21-30 years and only 9.49% of them have professional pharmacy education. They also concluded that at present the standard of community pharmacy was highly un-satisfactory. More pharmacists need to be involved at community pharmacy level besides developing awareness programmes to solve patient’s issues related to drugs to reduce the disease burden from the society. In the real world pharmacy in the developing low resource countries, private sector is dominant player in providing pharmacy services since Governments cannot afford to establish, man and run the drug stores all over the country for various reasons. This provision of drugs through the private sector is associated with risks regarding availability, affordability as well as rational use and quality of drugs.^4^ Regulatory compliance remains a major challenge for the authorities which are often outstripped by the phenomenal growth of the private sector. The concept of Good Pharmacy Practices (GPP) seen in the developed world remains a dream in the low resource countries. The quality of pharmacy service deteriorates further if one goes to the city slums or far flung rural areas, which makes immediate interventions by the drug regulatory authorities essential to ensure rational use and avoid misuse of drugs.

Yet another study from Pakistan looked at the role of pharmacists in developing countries which showed that services of qualified pharmacists are still underutilized and their role as healthcare professional was not considered important by the community as well as the medical profession including healthcare providers.^5^ Pharmacy profession in Pakistan is evolving gradually and country’s healthcare system has yet to recognize their role. In a country like Pakistan which is the case with many other low resource countries, a pharmacist not only has to be competent as regards pharmaceuticals but he or she also has to perform the role of a Manager particularly if they have to work at pharmacies established with the healthcare facilities. Community pharmacy run and managed by qualified pharmacists can play a vital role in several aspects of Good Pharmacy Practices and Rational Use of Drugs. But this objective cannot be achieved unless we have qualified pharmacists running and managing the retail chemists shops and pharmacies but even a study from Lao Peoples Democratic Republic showed that only 34% of pharmacies in public sector had pharmacists and its number was just negligible 4% in the private pharmacies.^6^

Yet another study from UAE regarding quality assessment of community pharmacy services showed that communication quality and different other aspects of service delivery quality were just more than average despite the fact that UAE is not a poor country.^7^ A study from Nepal regarding assessment of regulatory compliance in selected pharmacy outlets also showed that most of the outlets failed to comply with the regulatory provisions of Drugs Act 1978 and Codes on Sales and distribution of Drugs 2014.^8^ All this shows that the system in all these countries cannot be improved till meaningful reforms are introduced in the health services recognizing and extending the community pharmacists role to improve pharmacy services and meet patient’s demand.

There is no denying the fact that reforms and interventions are needed in different areas. While we need to introduce, promote and popularize the concept of community pharmacy, the regulatory compliance was also extremely important. For that those entrusted with this task i.e. Drug Inspectors and others involved in regulatory compliance need to be properly trained. Just pharmacy degree was not enough. They should be given comprehensive training before induction and also provided incentives, facilities to perform their duties. Only then they will get some respect in the community and the health services. The pharmacists also need to improve their Attitudes, Behaviour and Communication Skills if they wish that the medical profession and health establishments give them due respect and they can gain the status of being important member of healthcare team, something which the pharmacists enjoy in the developed world. Once they improve their professional skills and capabilities, the doctors will start listening to them and give importance to their advice and suggestions. When doctors and pharmacists start working as a team it will not only improve healthcare but also lead to minimizing the chances of any medical errors, undesirable adverse events related to drugs, drug interactions, thus reducing the period of hospitalization as well as overall healthcare cost which should remain the ultimate objective.

During the last few years a large number of new pharmacy institutions have been established with the result that there is no dearth of manpower as regards pharmacy graduates. However, a vast majority of these fresh pharmacy graduates just like medical graduates are girls and under the present set up and environment they cannot be expected to work at drug stores. More recently, we see different chains of pharmacy outlets being established by the private sector in major cities and one hopes with the passage of time their number will also increase. Since they are being established, run and managed by either pharmacists themselves or businessmen, they do have much better working environment, facilities which can absorb some of these fresh girl pharmacy graduates. However, the real progress will be made when the government decides to have qualified pharmacy graduates at all its healthcare facilities running pharmacy services.
